# Leather, Textiles and Bio-Based Materials

**DOI:** 10.3390/ma19194122

**Published:** 2026-09-26

**Authors:** Virgilijus Valeika

**Affiliations:** Department of Polymer Chemistry and Technology, Faculty of Chemical Technology, Kaunas University of Technology, Radvilenu pl. 19, 50254 Kaunas, Lithuania; virgilijus.valeika@ktu.lt

## 1. Introduction

Since time immemorial, materials of animal or plant origin, such as leather, wool, silk or cotton, flax or hemp fibres, and fabrics made from them, as well as other materials of biological origin, have remained extremely important because of their unique properties, which synthetically produced materials cannot replicate. Bio-based materials are characterized by excellent biocompatibility with the human body [1,2]; they are biodegradable [3]; their waste products are generally not harmful to the environment [4]; and they are derived from renewable sources [5].

For example, we can tan the collagen of hide/skin and use the produced leather for clothing, footwear, furniture or car upholstery—leather has a vast range of applications [6]. Also, we can produce gelatine from the collagen and eat it [7]. It can be used in various medical [8] or cosmetic preparations [9].

Although much is known about leather, wool, hemp, cotton, cellulose and other bio-based materials, the abundance of research and reports on their properties suggests that current knowledge is not yet comprehensive and that there may be new ways of producing, utilizing, and managing waste from these materials.

## 2. Overview of Published Articles

This Special Issue was dedicated to original contributions exploring the processing, properties, structures, and uses of bio-based materials for various purposes. Topics of interest included process development, structural changes, mechanical characterization, exploitation properties, and analytical and numerical methods to model, simulate, and predict the behavioural properties of the materials and products produced. The ten articles featured in this Special Issue represent a diverse spectrum of bio-based materials research.

### 2.1. Impact of Textile Components on the Properties of Textile Products and on Environmental Pollution

Even before the recorded history of humans, biopolymer fibres were employed for textile apparatus [10]. Textile materials were made from natural fibres such as wool, linen, cotton and silk, and they were used primarily for clothing. Currently, a huge amount of textiles is made from synthetic petroleum-based fibres, which has adverse impacts on the environment [11]. Due to the various problems associated with the use of petroleum-based materials and the growing recognition that textiles made from synthetic materials lack many of the properties of natural fibres, such as biocompatibility with the human body, sweat and moisture permeability, etc., there is a renewed interest in natural materials [10]. On the other hand, fibre blending for the manufacturing of textiles plays a crucial role in modern textile manufacturing. Usually, different natural and synthetic fibres are blended to achieve better qualities of both natural and synthetic materials [12].

In their study, Zhou et al. (Contribution 3) presented results of an investigation of properties of different types of commercially available running socks: regular plain running socks made of bio-based (cotton, wool) and/or synthetic materials (polyamide, polyester, and elastane) and specialized running socks, which are blended from high-performance bio-composite fibres (mohair, elastane, and nylon). The experiments included compression, tensile and shock absorption tests, whilst the wear tests assessed the load on the soles of the feet experienced by 10 adult amateur runners on a running track.

This study showed that the differences in the shock-absorbing properties between regular and specialized running socks, as confirmed by material testing, were primarily reflected in the maximum force absorption coefficient, which correlated directly with fabric thickness. Furthermore, compared with standard running socks, the specialized running socks produced lower average peak pressure in the big toe and first metatarsal areas, as well as a shorter forefoot ground-contact duration during running. These effects were attributed to the different toe designs of the socks and the properties of the fibre material. These effects were attributed to sock toe design variations and fibre material characteristics. According to the authors, the study offers new insights into how to optimize running socks, thereby promoting foot health in sports.

The results of similar studies are presented in the article by Kwan et al. on the influence of textiles and their components on the properties of sports bras (Contribution 5). The study examined the design of a flexible, form-fitting sports bra that utilized advanced knitting technologies and bio-based materials, including organic cotton and renewable acetate, to improve comfort and adaptability. This adaptive, seamless sports bra offered excellent breathability and was fully adjustable to accommodate changes in body shape, while ensuring comfort and functionality; the integrated spacer fabric provided excellent support and durability, while the “pointelle” structure enhanced breathability and moisture management. The insights presented in this article offer guidance on designing sports bras that better meet the needs of active teenagers.

Pešič et al. dedicated their research to the assessment of pineapple bio-based nonwoven textile as a sustainable alternative to natural leather and synthetic polymer-coated materials (Contribution 4). The material was produced from agricultural waste generated after the pineapple harvest by preparing pineapple leaf fibres (PALF) [13] and combining them with corn-based polylactic acid (PLA). The resulting material was mechanically treated and coated either with a mixture of bio-polyurethane (bio-PU) and conventional PU, or with conventional PU. During the investigation, both the objective properties of the material and subjective user feedback obtained while wearing a skirt made from the material were analyzed. The results of the study revealed a complex relationship between the subjective perception of comfort and objective physiological and thermal parameters. The results of the study showed that, when assessed using both subjective and objective indicators, the sample covered by a mixture of bio-PU and PU was rated more favourably in terms of thermophysiological comfort compared with the sample covered only by PU.

According to the authors, the garments made from bio-based material can be used for fashion items intended for indoor wear; however, mainly for shorter periods and special occasions. Prolonged wear of this material is not ideal due to a more pronounced sensation of thermal discomfort. Given the material’s thermophysiological properties, further research should test the potential applications of pineapple textile in garments designed for outdoor wear in colder climates, where its heat-retention ability could be an advantage.

Gomes et al. incorporated banana leaf extract into knitted hemp fabrics by soaking them in the extract to impart antimicrobial properties to the textile (Contribution 1). Studies have shown that the antimicrobial and antioxidant properties of the textiles were influenced by the method used to obtain the banana extract for the treatment. Antioxidant activity was highest in knits treated with ultrasound-assisted extracts using 50% ethanol as the extraction solvent, while the antimicrobial properties were most pronounced in knits treated with hydroalcoholic extracts obtained using pressurized methods. These extracts enhanced UV protection, with knits treated with 50% ethanol extracts via ultrasound-assisted extraction showing the highest UV Protection Factor. The results obtained provided insights for the development of innovative functional textile products with enhanced bioactive properties, suitable for a wide range of applications in both the textile and healthcare sectors.

A composite textile made from polypropylene and woven willow wood was studied by von Boyneburgk et al. (Contribution 2). They explored the influence of various climatic conditions, such as kiln-drying, frost, standard and tropical climatic conditions, artificial weathering and immersion in water, on the chemical and mechanical properties of the composite. The results showed that thermo-hygroscopic influences, particularly during weathering, significantly affected the textile properties, mainly because of the wood’s hygroscopic nature. Most of the mechanical properties were negatively affected by both increases and decreases in moisture content; however, this trend did not apply to all characteristics, as some properties even showed improvements under certain ageing conditions.

The question arises as to how these findings might be applied in practice, given that the phenomena under investigation rarely occur in isolation but usually occur simultaneously or in succession. The main conclusion was that the thermo-hygroscopic effects studied contribute to the medium- to long-term degradation of the material and that stabilization (by sealing exposed wooden edges to prevent moisture ingress and/or by stabilizing the polymer matrix using UV stabilizers) against weather-induced degradation is essential.

Although materials of biological origin are considered environmentally friendly, their treatment processes often require large amounts of water and chemicals, resulting in contaminated effluent and other pollutants. The production of textiles from fibres is no exception. Textile manufacturing uses various chemicals, including pesticides, dyes, auxiliaries, and polyfluorinated alkyl compounds, which alter fibre composition, affect degradation properties, and increase the environmental risks associated with microfibre release [14].

In a brief review article, Hossain et al. [15] assessed the release of microplastics at various stages of textile production. The authors stated that the textile industry contributes significantly to microplastic (MP) pollution, particularly in the production of synthetic fibres and blends of natural and synthetic fibres, as large quantities of fibrous MPs are released during manufacturing; these account for approximately 49–70 percent of all MPs found in wastewater worldwide, and are mainly generated during textile production and the domestic laundering of synthetic fabrics. The article focuses on the processes most influential in MP shedding, including spinning, weaving, and knitting in the dry phase, and bleaching, scouring, dyeing, printing, and finishing in the wet phase. Given the increasingly widespread use of textile products across various areas, the scale of microplastic pollution extends beyond the boundaries of traditional areas of use, and it is, therefore, essential to urgently address the impact of fibre-based microplastic release from synthetic and bio-based textile products. According to the authors, the development of technologies designed to reduce microplastic release during textile production represents a promising approach to tackling this serious environmental issue.

### 2.2. Improvements in Leather Processing

Leather processing involves a series of complex processes that purposefully alter the structure of the skin’s/hide’s dermis and remove substances that are undesirable in terms of leather quality. Unfortunately, the leather industry suffers from negative impacts due to environmental pollution caused by tannery wastes produced during leather processing steps [16]. The research presented by Biškauskaitė-Ulinskė and Valeika was dedicated to a new pickling process using *p*-toluenesulfonic acid monohydrate (*p*TsOH) instead of sulfuric acid and sodium chloride (Contribution 6). *p*TsOH is a strong acid and oxidizer and, therefore, acts accordingly; however, it is not classified as a carcinogenic, mutagenic, or teratogenic material or reproductive toxicant. The pickled hide obtained after experimental pickling showed a higher shrinkage temperature, while the collected solutions contained lower amounts of TDS, TSS, and chlorides. During chrome tanning, the new technology showed further advantages: chromium compound exhaustion exceeded 98% compared with only 68.7% after conventional pickling, and the amount of chromium compounds in the leather was much higher than in the control sample. Consequently, smaller amounts of basic chromium sulphate could be used during chrome tanning to achieve the same chromium content in the leather. The properties of the crust leather produced after experimental pickling were comparable to those of the conventionally produced one.

Another way to improve the properties of leather is to treat it by physical or chemical methods [17]. Gaidau et al. (Contribution 8) treated leather surfaces with TiO_2_-based nanocomposites decorated with silver (Ag), copper oxides (Cu_2_O/CuO) and graphene oxide. The nanoparticles were applied to the leather surface in finishing films and subsequently activated by gamma irradiation at 25 kGy. This treatment ensured long-lasting antimicrobial resistance of the leather. This proposed methodology is compatible with industrial leather processing and demonstrates that leather with antimicrobial protection can be produced for use in biosafety products.

### 2.3. Hemp for Heat Isolation and for Biomass

This Special Issue contains two papers on the use of hemp for technical purposes. This biological material has been unjustly neglected for some time, perhaps due to the misattribution of certain properties, but recently, research related to the application of hemp has clearly been on the rise.

Vėjelis et al. investigated [18] the thermal insulation properties of hemp, hemp-wool blends, and wool-based materials. It should be noted that wool has long been known as a thermal insulation material, but its properties depend heavily on its origin [19]. For the prepared specimens, the dependence of the thermal conductivity (varied within the range of 0.0333 ÷ 0.0438 W/(m·K)), the tensile strength (from 2.5 to 130 kPa) and the short-term water absorption (from 0.5 to 8.5 kg/m^2^) on the density of the specimens and the flammability parameters were determined. The use of hydrophobic agents and flame retardants can significantly reduce the water absorption and flammability values. According to the authors, the obtained results had expanded the spectrum and data of research on the composition and properties of natural fibre-based thermal insulation materials and created additional opportunities to choose appropriate materials.

Industrial hemp has excellent agronomic and pharmaceutical characteristics, and its valorization in various aspects is highly required. Several representative pretreatment technologies used for hemp biomass are proposed in terms of sugar recoveries, lignin removal, and sugar and ethanol yields [20]. Pretreatment is necessary to access cellulose by removing lignin, hemicellulose, and extractives (Contribution 7). Myeni et al. investigated three hemp hurds’ pretreatment methods: organo-solvent, oxidative-alkaline, and sonication, to improve bioconversion of hemp and to find dependencies between changes in biomass caused by pretreatment and changes in functional bioconversion results. The data obtained led to the conclusion that NaOH-based pretreatment resulted in more identified changes in polysaccharide content of 48.5%, crystallinity index of 65.80%, and thermal behaviour. Hemp hurds pretreated this way exhibited the most disrupted surface, indicating substantial modification in their physical structure. The authors emphasized that the obtained results provide guidance on how to select the appropriate pretreatment for hemp processing.

## 3. Conclusions

This Special Issue brings together ten articles covering a wide range of topics; they present important research that is expected to pave the way for the obtaining and processing of bio-based materials, the manufacture of products from them, and the utilization of their waste, using safe and sustainable production processes, with a view to addressing environmental challenges in a world that needs to reduce its dependence on oil. These studies promote the development of advanced processing technologies for bio-based materials and the expansion of their areas of application while offering new strategies for processing these materials and manufacturing products from them.

The presented contributions will be of particular interest to processors, users, and consumers of bio-based materials, especially those who use these materials in the textile, clothing, and building sectors, with a view to bringing more environmentally friendly materials to market in the future.
